# The Effect of D-(−)-arabinose on Tyrosinase: An Integrated Study Using Computational Simulation and Inhibition Kinetics

**DOI:** 10.1155/2012/731427

**Published:** 2012-12-23

**Authors:** Hong-Jian Liu, Sunyoung Ji, Yong-Qiang Fan, Li Yan, Jun-Mo Yang, Hai-Meng Zhou, Jinhyuk Lee, Yu-Long Wang

**Affiliations:** ^1^Zhejiang Provincial Key Laboratory of Applied Enzymology, Yangtze Delta Region Institute of Tsinghua University, Jiaxing 314006, China; ^2^Korean Bioinformation Center (KOBIC), Korea Research Institute of Bioscience and Biotechnology, Daejeon 305-806, Republic of Korea; ^3^Department of Bioinformatics, University of Sciences and Technology, Daejeon 305-350, Republic of Korea; ^4^Department of Dermatology, Sungkyunkwan University School of Medicine, Samsung Medical Center, Seoul 135-710, Republic of Korea

## Abstract

Tyrosinase is a ubiquitous enzyme with diverse physiologic roles related to pigment production. Tyrosinase inhibition has been well studied for cosmetic, medicinal, and agricultural purposes. We simulated the docking of tyrosinase and D-(−)-arabinose and found a binding energy of −4.5 kcal/mol for theup-formof D-(−)-arabinose and −4.4 kcal/mol for thedown-form of D-(−)-arabinose. The results of molecular dynamics simulation suggested that D-(−)-arabinose interacts mostly with HIS85, HIS259, and HIS263, which are believed to be in the active site. Our kinetic study showed that D-(−)-arabinose is a reversible, mixed-type inhibitor of tyrosinase (*α*-value  = 6.11 ± 0.98, *K*_*i*_ = 0.21 ± 0.19 M). Measurements of intrinsic fluorescence showed that D-(−)-arabinose induced obvious tertiary changes to tyrosinase (binding constant *K* = 1.58 ± 0.02 M^−1^, binding number *n* = 1.49 ± 0.06). This strategy of predicting tyrosinase inhibition based on specific interactions of aldehyde and hydroxyl groups with the enzyme may prove useful for screening potential tyrosinase inhibitors.

## 1. Introduction

Tyrosinase (EC 1.14.18.1) is a ubiquitous enzyme with diverse physiologic roles related to pigment production. It plays a central role in melanin synthesis in skin [1, 2], the browning of vegetables [3, 4], wound healing [5], and cuticle formation in insects [6, 7]. Structurally, tyrosinase belongs to the type 3 copper protein family [8, 9], which consists of two copper ions individually coordinated with three histidine residues at the active site. Tyrosinases are directly involved in several reactions and carry out catalytic steps such as the hydroxylation of tyrosine to 3,4-dihydroxyphenylalanine (DOPA), the oxidation of DOPA to DOPA quinone, and the oxidation of 5,6-dihydroxyindole to 5,6-dihydroxuquinone [10, 11]. In addition to its catalytic features, tyrosinase is distinctive from other enzymes because it displays various inhibition patterns. Tyrosinase inhibition has been extensively studied for cosmetic, medicinal, and agricultural purposes [12].

The tyrosinase mechanism is complex, and this enzyme can catalyze multiple reactions. Despite several reported crystallographic structures of tyrosinase, the 3D structure and architecture of the active site are not well understood [22, 23]. Mechanistic studies must involve a variety of computational methods and kinetic analysis to derive the structure-function relationship between substrates and ligands. The inhibitory effect of compounds with sugar backbones on tyrosinase are of great interest [20, 24, 25]. D-(–)-arabinose, a potential tyrosinase inhibitor, is an aldopentose with one aldehyde and four hydroxyl groups, which was used to immobilize mushroom tyrosinase on a reusable glass bead preparation [26].

In the current study, we investigated the mechanism of tyrosinase inhibition by D-(–)-arabinose using computational simulation and kinetic analysis. We hypothesized that the aldehyde and hydroxyl groups of D-(–)-arabinose may block L-DOPA oxidation by binding to tyrosinase. Previous findings have shown the importance of aldehyde [27, 28] and hydroxyl [27, 29–32] groups in tyrosinase inhibition with regard to molecular position, number, and specific interactions of these groups with the enzyme. These findings further support our hypothesis that D-(–)-arabinose might have an inhibitory effect on tyrosinase, as D-(–)-arabinose has one aldehyde and four hydroxyl groups. D-(–)-arabinose exerted a mixed-type inhibition on tyrosinase. Kinetic parameters have consistently supported docking simulation results in which D-(–)-arabinose binds to residues at or near the active site, and measurements of intrinsic fluorescence have revealed great changes in tertiary protein structure. A combination of computational modeling and inhibition kinetics may facilitate the testing of potential tyrosinase inhibitors such as D-(–)-arabinose and the prediction of their inhibitory mechanisms.

## 2. Materials and Methods

### 2.1. Materials

Tyrosinase (M.W. 128 kDa) and L-DOPA were purchased from Sigma-Aldrich. D-(–)-arabinose was purchased from Tokyo Chemistry Industry. When L-DOPA was used as a substrate in our experiments, the purchased tyrosinase had a *K*_*m*_ of 0.29 ± 0.11 mM (*V*_max⁡_ = 0.15 ± 0.03 mmol · min⁡^−1^) using a Lineweaver-Burk plot. All kinetic reactions and measurements in this study were performed in 50 mM sodium phosphate buffer (pH 6.9) at the temperature of 25°C.

### 2.2. Computational Docking of Tyrosinase and D-(–)-arabinose and Molecular Dynamics Simulation

The tyrosinase structure was simulated according to the crystal structure of *Agaricus bisporus* tyrosinase (PDB ID: 2Y9X). The original structure of D-(–)-arabinose was derived from the PubChem database (ID: 66308), named (2S, 3R, 4R)-2, 3, 4, 5-tetrahydroxypentanal. At room temperature, D-(–)-arabinose exists in a ring structure because the terminal alcohol and aldehyde group interact with each other. Because the structure has an ambiguous chiral center in the ring, we generated two chemical forms (up and down). We used the Pck software to find the binding pocket [33] and found several neighboring residues from the binding pocket. Ten docking structures were generated from each neighboring residue. AutoDock Vina [34] was used for* in silico* protein-ligand docking. Using the final structure from the docking result, a 10 ns production of molecular dynamics simulation was performed by CHARMM [35]. Then, we measured the structure details of the protein-ligand interactions as a function of time to ensure that the interactions revealed by the docking study were conserved. The structures were saved every picosecond for trajectory analysis.

### 2.3. Tyrosinase Assay

A spectrophotometric tyrosinase assay was performed as previously described [16, 18, 36]. To begin the assay, a 10 *μ*L sample of enzyme solution was added to 1 mL of reaction mix. Tyrosinase activity (*υ*) was recorded as the change in absorbance per min at 475 nm using a Helios Gamma spectrophotometer (Thermo Spectronic, UK). The final concentrations of L-DOPA and tyrosinase were 2 mM and 6.6 *μ*g/mL, respectively. A range of D-(–)-arabinose from 0.05 M to 2 M was applied, according to the experimental conditions.

### 2.4. Kinetic Analysis for the Mixed-Type Inhibition

To describe the mixed-type inhibition mechanism, the Lineweaver-Burk equation in double-reciprocal can be written as follows:

(1) $$\begin{array}{c} \frac{1}{\upsilon }=\frac{K_{m}}{V_{\max}}\left(1+\frac{\left[I\right]}{K_{i}}\right)\frac{1}{\left[S\right]}+\frac{1}{V_{\max}}\left(1+\frac{\left[I\right]}{\alpha K_{i}}\right). \end{array}$$

Secondary plots can be constructed from

(2) $$\begin{array}{c} \text{Slope}=\frac{K_{m}}{V_{\max}}+\frac{K_{m}\left[I\right]}{V_{\max}K_{i}}, \end{array}$$

(3) $$\begin{array}{c} Y\text{-intercept}=\frac{1}{V_{\max}^{\text{app}}}=\frac{1}{V_{\max}}+\frac{1}{{\alpha K}_{i}\;V_{\max}}\left[I\right]. \end{array}$$

**Scheme 1:**
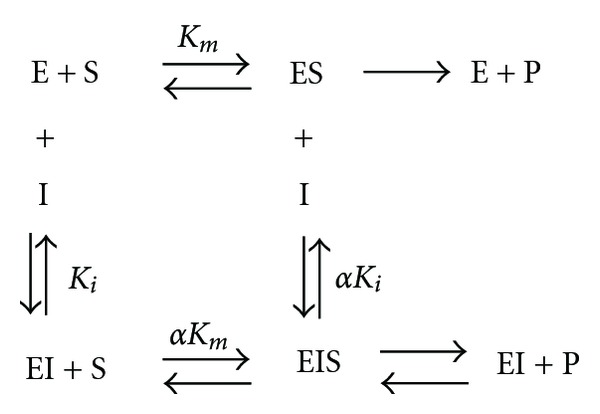
D-(–)-arabinose binds to the E and ES complex. E: enzyme tyrosinase; S: substrate L-DOPA; I: inhibitor D-(–)-arabinose; P: product dopachrome; *K*_*i*_: inhibitor dissociation constant; *α*: modifying factor.

The *V*_max⁡_, *K*_*m*_, *α*, and *K*_*i*_ values can be derived from the above equations. The secondary plot of Slope or *Y*-intercept versus [*I*] is linearly fit, assuming a single inhibition site or a single class of inhibition site, as shown in Scheme 1.

### 2.5. Intrinsic and ANS-Binding Fluorescence Measurements

Fluorescence emission spectra were measured using a Hitachi F-2500 fluorescence spectrophotometer with a 1 cm path-length cuvette. An excitation wavelength of 280 nm was used for the tryptophan fluorescence measurements, and the emission wavelength was between 300 and 420 nm. Changes in the ANS-binding fluorescence intensity for tyrosinase were studied by labeling with 40 *μ*M ANS for 30 min prior to measurement. An excitation wavelength of 390 nm was used for the ANS-binding fluorescence, and emission wavelength ranged from 420 to 600 nm. The final concentrations of tyrosinase was 6.6 *μ*g/mL.

### 2.6. Determination of the Binding Constant and the Number of Binding Sites

According to a previous report [37], when small molecules are bound to equivalent sites on a macromolecule, the equilibrium between free and bound molecules is given by the following equation to evaluate the binding constant (*K*) and number of binding sites (*n*):

(4) $$\begin{array}{c} \frac{F_{0}}{F_{0}-F}=\frac{1}{n}+\frac{1}{K}\frac{1}{\left[Q\right]}, \end{array}$$

where *F*_0_ and *F* are the relative steady-state fluorescence intensities in the absence and presence of quencher, respectively, and [*Q*] is the quencher (D-(−)-arabinose) concentration. The values for the binding constant (*K*) and number of binding sites (*n*) can be derived from the intercept and slope of a plot based on (4).

## 3. Results

### 3.1. Computational Docking and Molecular Dynamics Simulation

**Figure 1:**
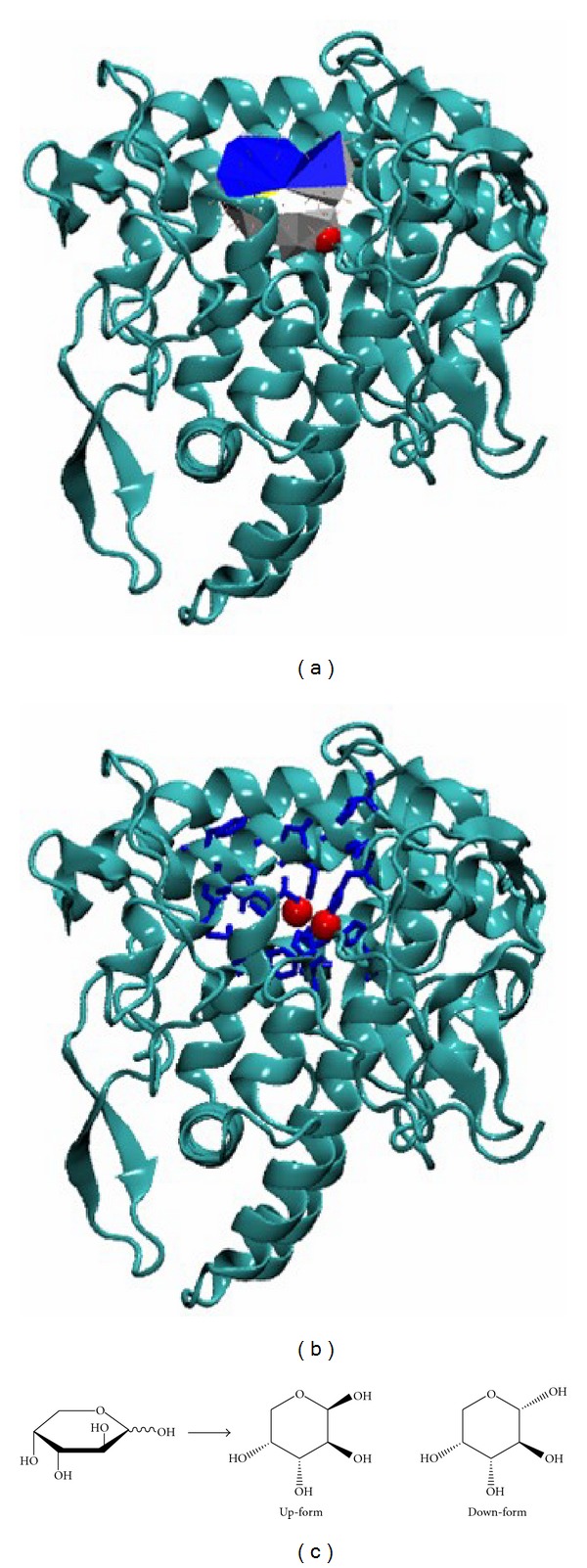
Computational simulations of tyrosinase and D-(–)-arabinose. (a) The binding pocket of tyrosinase was found using Pck software. Blue and white represent the pocket mouth, with the entrance colored in blue. The two red spheres indicate the two copper ions. (b) Plausible binding residues. Blue sticks indicate the pocket residues, and the two red spheres indicate the two copper ions. (c) The two forms of D-(–)-arabinose (up-form and down-form).

We searched for the D-(−)-arabinose binding pocket (Figure 1(a)) and common neighboring residues within tyrosinase and found several residues (Figure 1(b)), including HIS61, HIS85, HIS94 HIS244, GLU256, HIS259, ASN260, HIS263, PHE264, MET280, GLY281, SER282, VAL283, ALA286, PHE292, and HIS296. As the 1′-OH group of D-(−)-arabinose is flexible, there are two forms that exist: up and down (Figure 1(c)). The lowest docking energies of up-form and down-form D-(−)-arabinose were −4.5 kcal/mol and −4.4 kcal/mol, respectively. The negative energies indicated that the two chemicals were bound tightly to tyrosinase. Thermodynamically, the interactions between up-form D-(−)-arabinose and tyrosinase could be more quickly stabilized than those in down-form.

**Figure 2:**
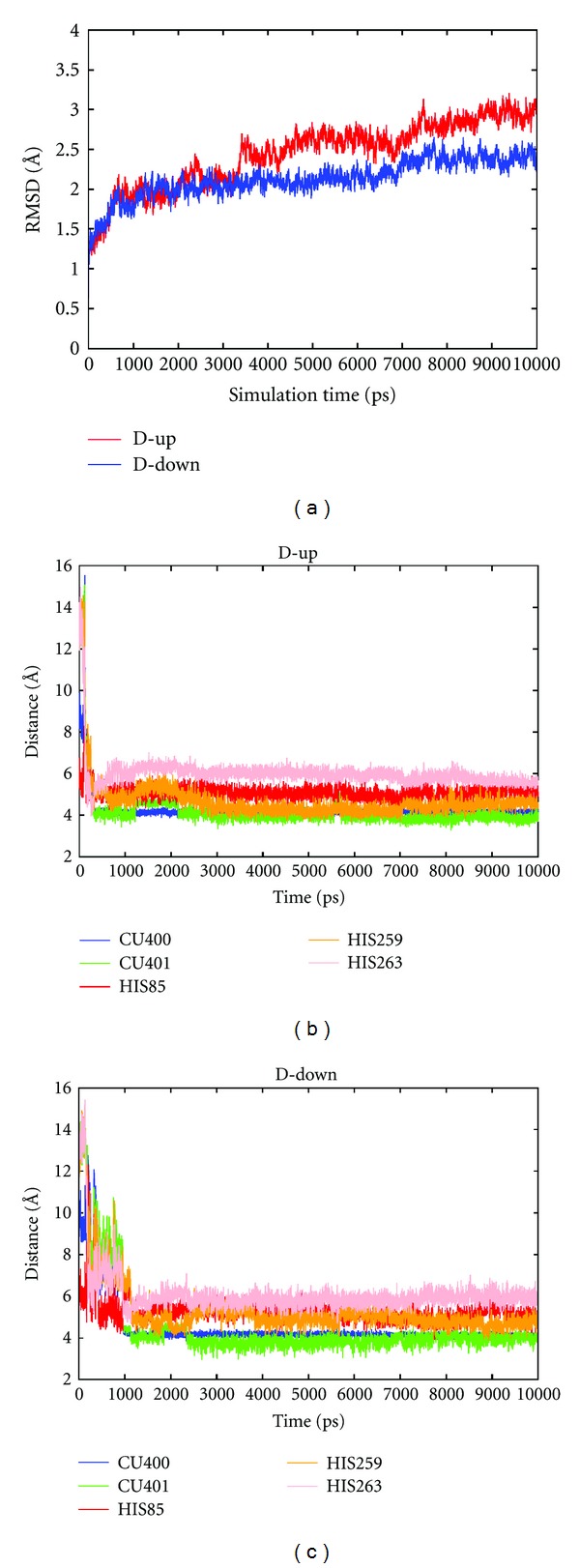
Molecular dynamics simulations between tyrosinase and D-(–)-arabinose. (a) RMSD time profiles of the alpha carbon. (b) Time profile of plausible interactions with up-form D-(–)-arabinose. (c) Time profile of plausible interactions with down-form D-(–)-arabinose. Cu400 and Cu401 represent the two copper ions.

**Figure 3:**
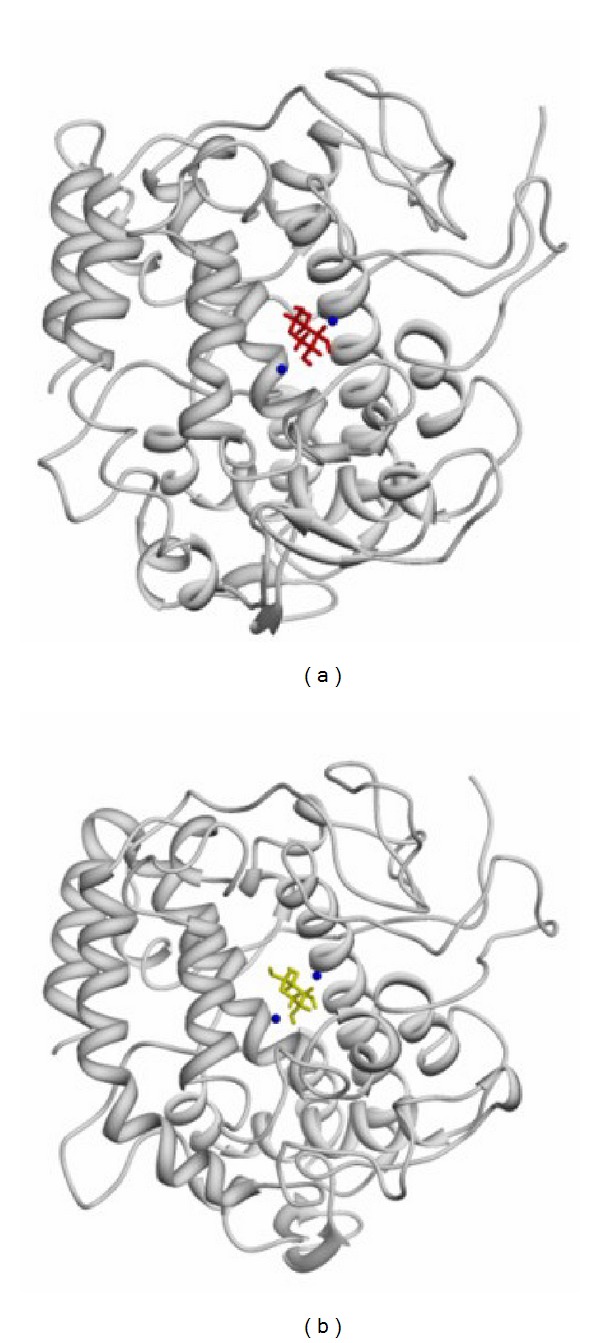
Binding regions predicted by molecular dynamics simulations. (a) Binding site for up-form D-(–)-arabinose. (b) Binding site for down-form D-(–)-arabinose.

Next, the 10 ns molecular dynamics simulation was performed to examine structural changes during simulation. We verified the root mean square deviation (RMSD) of the alpha carbon, and the results showed that the structure was first rearranged and then stabilized (Figure 2(a)). Plausible candidate residues that may interact with D-(−)-arabinose were chosen, and the distances were measured via simulation. The interactions gradually stabilized after 1 ns as the simulation progressed (Figures 2(b) and 2(c)). The plausible residues determined by the molecular dynamics simulation were HIS85, HIS259, and HIS263 for both up-form (Figure 2(b)) and down-form (Figure 2(c)) D-(−)-arabinose. Both forms of D-(−)-arabinose were accessed to the core site of the pocket, and the binding of up-form D-(−)-arabinose with tyrosinase was slightly more stable than the down-form. The binding sites for both forms of D-(−)-arabinose with tyrosinase were predicted to be HIS85, HIS259, and HIS263 (Figure 3).

In our studies, the identified plausible residues for docking and the molecular dynamics simulations were consistent.

### 3.2. The Effect of D-(−)-arabinose on Tyrosinase Activity

**Figure 4:**
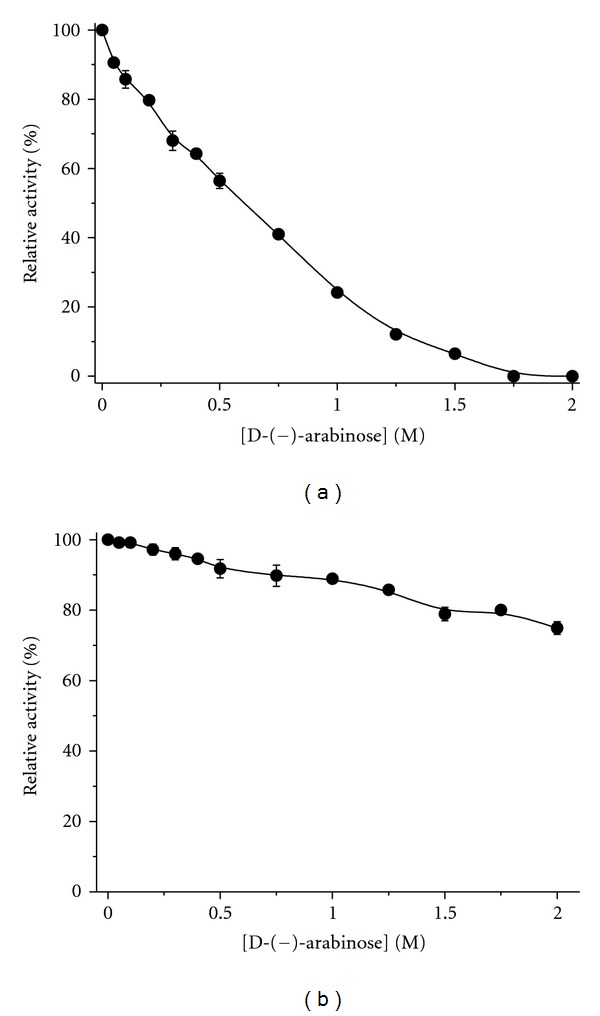
Inhibitory effect of D-(–)-arabinose on tyrosinase. Data are presented as the mean (*n* = 3). Tyrosinase was incubated with D-(–)-arabinose at various concentrations for 3 h at 25°C and then added to the assay system at the corresponding D-(–)-arabinose concentrations (a) or in the absence of D-(–)-arabinose (b). The final concentrations of L-DOPA and tyrosinase were 2 mM and 6.6 *μ*g/mL, respectively.

**Figure 5:**
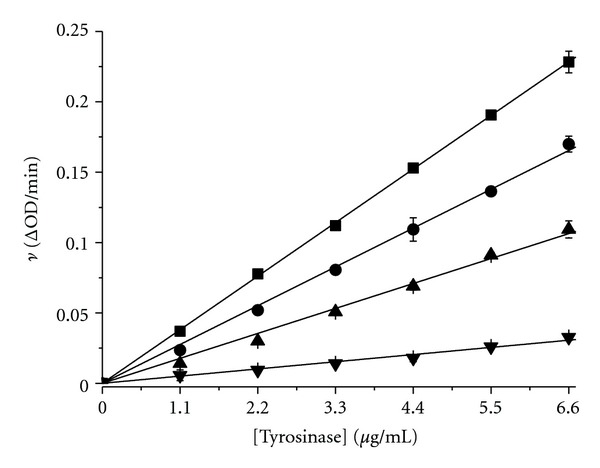
Plots of enzyme activity (*v*) versus enzyme concentration [E] at D-(–)-arabinose concentrations of 0 M (■), 0.2 M (●), 0.6 M (▲), and 0.8 M (*▼*). The *ν* value indicates the change in absorbance at 475 nm per minute. The final L-DOPA concentration was 2 mM.

We assayed tyrosinase activity changes in the presence of D-(−)-arabinose. Tyrosinase activity was inactivated by D-(−)-arabinose in a dose-dependent manner (Figure 4). The inhibitor concentration leading to 50% tyrosinase activity loss (IC_50_) was estimated to be 0.61 ± 0.02 M (*n* = 3), and tyrosinase was almost completely inactive at 1.75 M D-(−)-arabinose (Figure 4(a)). When D-(−)-arabinose was absent from the assay system, the tyrosinase activity remained at 76%, even with 2 M D-(−)-arabinose in the preincubation step (Figure 4(b)). The difference is due to the dilution effect of inhibitor, indicating that D-(−)-arabinose reversibly inhibited tyrosinase. To confirm the reversibility of D-(−)-arabinose inhibition, the remaining enzyme activity at different inhibitor concentrations was tested. A plot of enzyme activity (*v*) versus enzyme concentration [E] resulted in a decrease in the slope of the line, indicating that the inhibition of D-(−)-arabinose on the enzyme was reversible (Figure 5).

**Figure 6:**
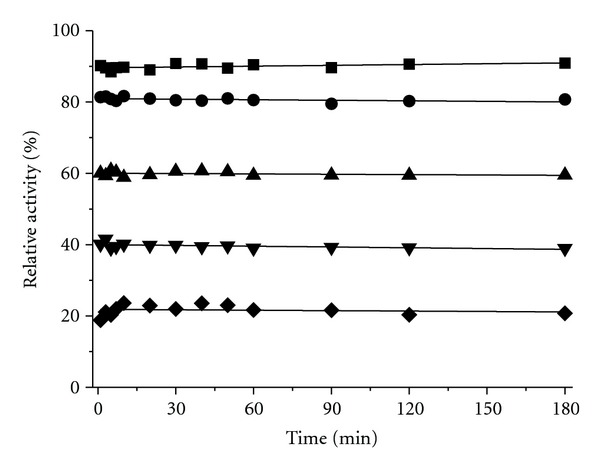
Time course of tyrosinase inhibition in the presence of D-(–)-arabinose. The enzyme solution was mixed with D-(–)-arabinose at 0.05 M (■), 0.2 M (●), 0.5 M (▲), 0.75 M (*▼*), and 1 M (◆) concentrations, and aliquots were collected for analysis at the indicated time intervals.

Next, time courses for tyrosinase inhibition at different concentrations of D-(−)-arabinose were performed (Figure 6). The results showed that changes in the catalytic rate over time were not detectable at any of the tested D-(−)-arabinose concentrations (0.05 M to 1 M). Thus, D-(−)-arabinose appeared to bind tyrosinase very quickly and inhibit the oxidation of L-DOPA without an apparent change in the catalytic rate under these test conditions. These results indicated that the overall tertiary structural changes might not be synchronized with D-(−)-arabinose-induced inhibition due to rapid achievement of equilibrium states.

### 3.3. Determination of the D-(−)-arabinose Inhibition Type

**Figure 7:**
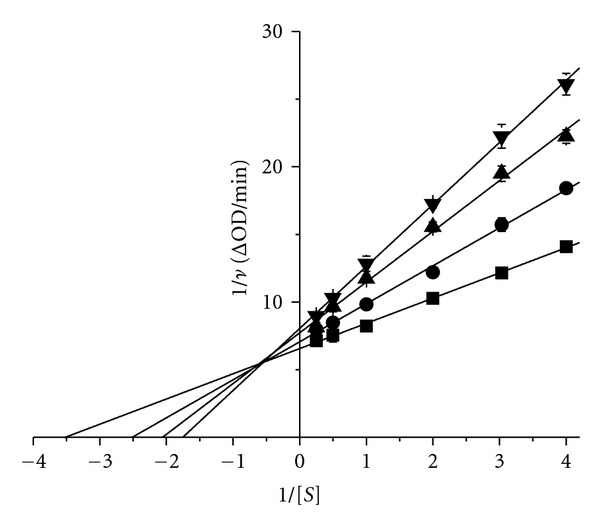
Lineweaver-Burk plot. The D-(−)-arabinose concentrations were 0 M (■), 0.1 M (●), 0.2 M (▲), and 0.3 M (*▼*). The final enzyme concentration was 6.6 *μ*g/mL.

**Figure 8:**
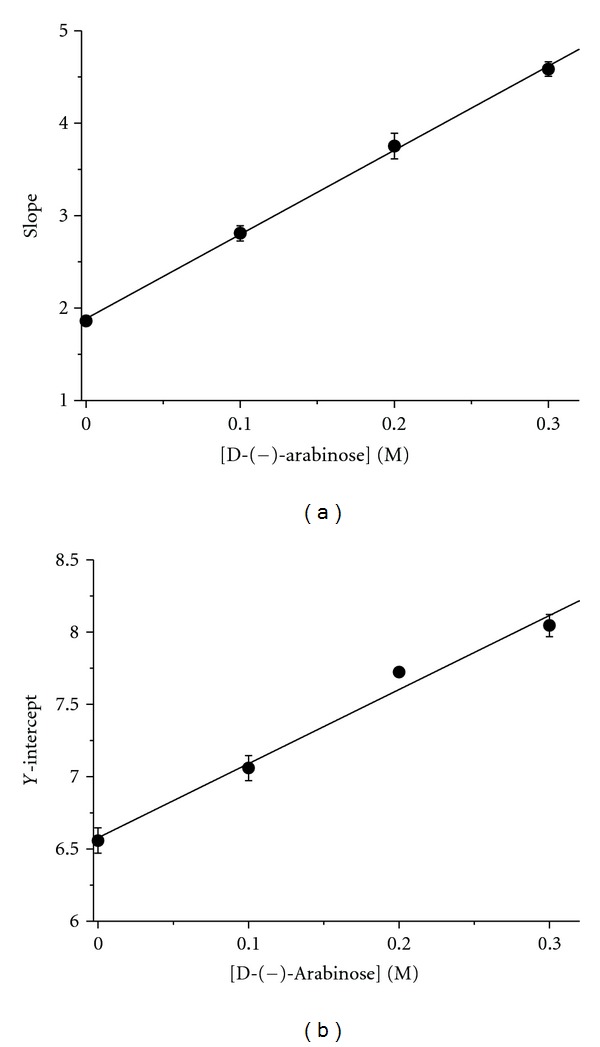
Secondary plots. (a) Plot of Slope versus D-(−)-arabinose concentration. All data were collected from Lineweaver-Burk plots. The plot was based on (2). (b) Plot of *Y*-intercept versus D-(−)-arabinose concentration. The plot was based on (3).

The kinetics of tyrosinase in the presence of D-(−)-arabinose was studied using double-reciprocal Lineweaver-Burk plots. The results showed changes in the apparent *V*_max⁡_ and the *K*_*m*_, indicating that D-(−)-arabinose induced a mixed-type of inhibition (Figure 7). The secondary plots of Slope versus D-(−)-arabinose concentration and *Y*-intercept versus D-(−)-arabinose concentration were linearly fit (Figures 8(a) and 8(b), showing that D-(−)-arabinose has a single inhibition site or a single class of inhibition site on tyrosinase. Using (1)–(3), the *α*-value was found to be 6.11 ± 0.98 (*n* = 3), and the *K*_*i*_ was 0.21 ± 0.19 M (*n* = 3).

### 3.4. The Effect of D-(−)-arabinose on Tyrosinase Tertiary Structure

**Figure 9:**
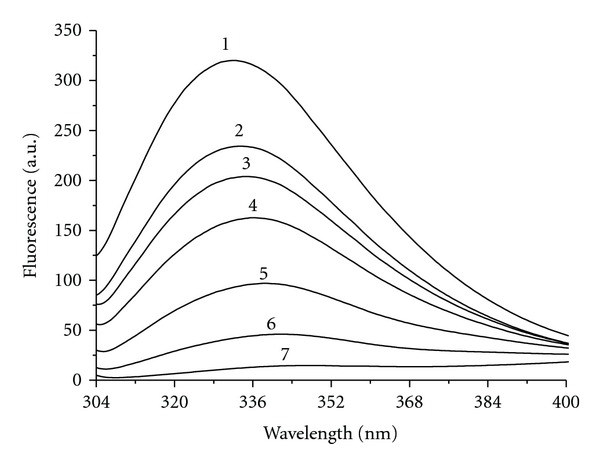
Intrinsic fluorescence changes by D-(−)-arabinose. Tyrosinase was incubated with D-(−)-arabinose for 3 h before the measurements were taken. Label 1 represents the native state. Labels 2 through 7 indicate D-(−)-arabinose concentrations of 0.2 M, 0.3 M, 0.5 M, 0.75 M, 1.25 M, and 2 M, respectively.

**Figure 10:**
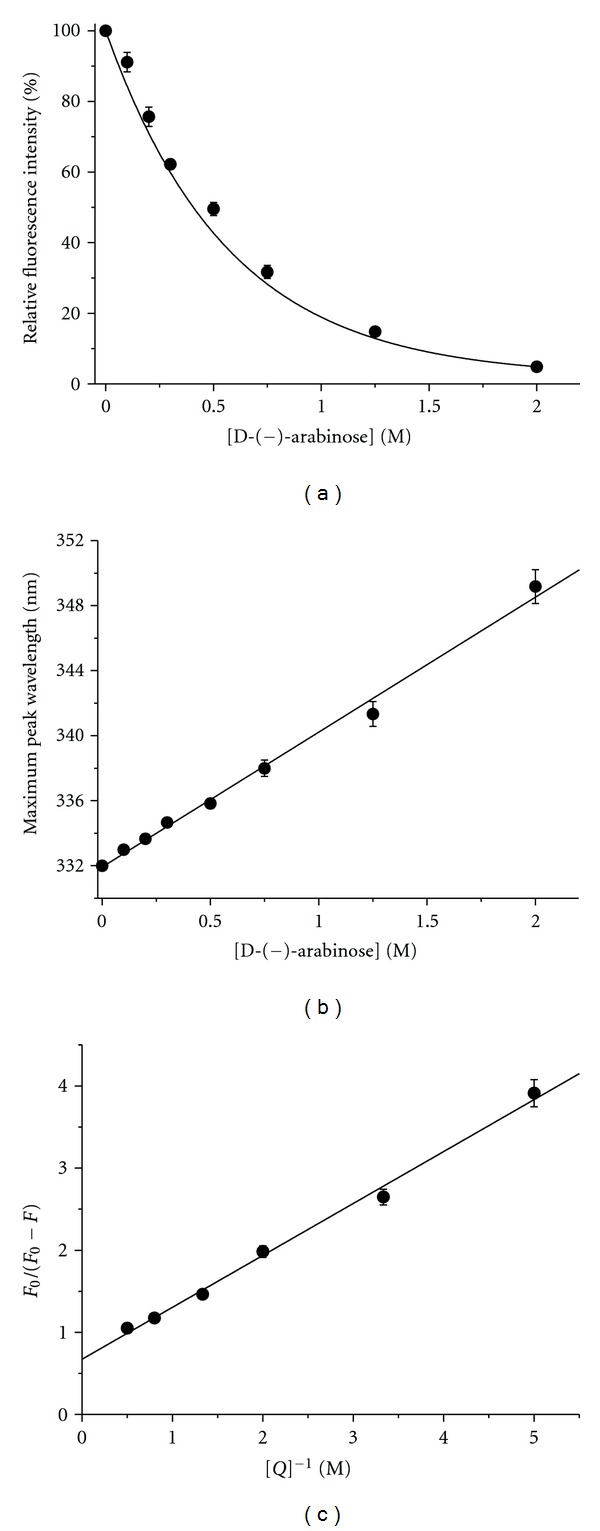
Tyrosinase tertiary structure changes in the presence of D-(−)-arabinose. Tyrosinase was incubated with various concentrations of D-(−)-arabinose (0 to 2 M). (a) Plot of maximum fluorescence intensity versus D-(−)-arabinose concentration. (b) Plot of maximum peak wavelength changes versus D-(−)-arabinose concentration. (c) Double reciprocal plot of *F*_0_/(*F*_0_ − *F*) versus [*Q*]^−1^. Data were treated according to (4). *F*_0_: maximum native fluorescence intensity; *F*: maximum fluorescence intensity of sample; *Q*: quencher D-(−)-arabinose.

Tertiary structural changes of tyrosinase induced by D-(−)-arabinose were determined from measurements of the enzymatic intrinsic fluorescence changes. We found that the intrinsic fluorescence of tyrosinase was significantly changed. This was accompanied by a quenching effect that gradually decreased, with a significant shift of the maximum peak wavelength from 332 nm to 349 nm (Figures 9, 10(a), and 10(b)). At a 2 M concentration, D-(−)-arabinose almost completely quenched the fluorescence. To calculate the binding affinity, a double-reciprocal plot was evaluated according to (4) as shown in Figure 10(c). The plot revealed a linear relationship, and we calculated the binding constant (*K* = 1.58 ± 0.02 M^−1^) and the binding number (*n* = 1.49 ± 0.06) according to (4).

**Figure 11:**
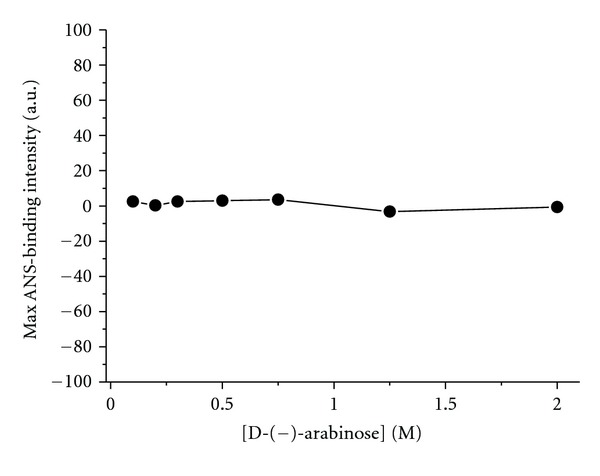
Changes in ANS-binding fluorescence of tyrosinase at different D-(−)-arabinose concentrations. ANS (40 *μ*M) was incubated with tyrosinase for 30 min to label the hydrophobic enzyme surfaces prior to fluorescence measurements.

Next, we monitored changes in tyrosinase hydrophobicity in the presence of D-(−)-arabinose (Figure 11). Unfortunately, the ANS-binding fluorescence of D-(−)-arabinose alone was so strong that we could not tell whether it had influence on the ANS-binding fluorescence of tyrosinase.

## 4. Discussion

Previous studies have identified compounds with aldehyde and hydroxyl groups to have a potent inhibitory effect on tyrosinase [38–40]. The hydroxyl groups in compounds carry out the nucleophilic attack on the coppers of tyrosinase in the active site and are directly involved in transferring protons during catalysis, which results in inactivation of tyrosinase [41, 42]. In this study, we hypothesized that D-(−)-arabinose could be a potent tyrosinase inhibitor due to its structure, with one aldehyde and four hydroxyl groups. 

Using *in silico *docking, we predicted that D-(−)-arabinose could bind directly with amino acid residues inside the tyrosinase active site. Further molecular dynamics simulation showed a specific binding site consisting of HIS85, HIS259, and HIS263 for the up- and down-forms of D-(−)-arabinose. We found that the enzymatic activity of tyrosinase decreased as the concentrations of D-(−)-arabinose increased. D-(−)-arabinose inhibited tyrosinase in a reversible way and induced changes in the apparent *V*_max⁡_ and the *K*_*m*_, consistent with a mixed-type of inhibition through interactions with residues at or close to the active site. The second plots of Slope versus D-(−)-arabinose concentration and *Y*-intercept versus D-(−)-arabinose concentration were linearly fit. These results were consistent with previous computational simulation. 

As conformational changes at or near the active site could influence access by the enzyme substrate, the conformational changes of tyrosinase induced by D-(−)-arabinose were detected by spectrofluorometry. Obvious changes were observed which indicated that the structure of the tyrosinase active site had been altered to reduce the access of the substrate L-DOPA. 

Changes in catalytic rate were not detectable over time, which indicated that D-(−)-arabinose binding to the enzyme and decreased enzyme activity occurred very quickly. These results indicated that the overall tertiary structural changes might not be synchronized with D-(−)-arabinose-induced inhibition of tyrosinase due to rapid achievement of equilibrium states. 

Our study had several principal findings: (1) D-(−)-arabinose binding to tyrosinase caused a mixed-type of inhibition; (2) D-(−)-arabinose inhibition of tyrosinase caused gross changes in tertiary structure, detected as unfolding; (3) putative D-(−)-arabinose-binding residues HIS85, HIS259, and HIS263 were predicted using computational simulation. Further molecular dynamics simulation predicted the specific binding site of HIS85, HIS259, and HIS263 for the up- and down-forms of D-(−)-arabinose.

Compared with other inhibitors, D-(−)-arabinose is a poor inhibitor of tyrosinase (Table 1). D-(−)-arabinose is less effective to tyrosinase than L-(−)-arabinose (*K*_*i*_ = 0.22 ± 0.07 mM) [15]. Therefore, our study directly provides critical information that simple change of configuration is critical for the inhibition of tyrosinase and the results can provide basical information for the further designing or screening of tyrosinase inhibitors.

## 5. Conclusion

The inhibitory mechanism of D-(−)-arabinose resembles that of a copper chelator but differs in that D-(−)-arabinose does not directly bind to the copper atoms in the active site. Our study provides fresh insight into the role of active site residues in tyrosinase catalysis, and presents useful information regarding the 3D structure of tyrosinase. Minor change in the configuration of arabinose can lead to major difference of the inhibition effect on tyrosinase. These findings provide important information in the study of tyrosinase inhibitors in the development of hyperpigmentation treatment. A combination of computational modeling and inhibition kinetics may facilitate the testing of potential tyrosinase inhibitors and the prediction of their inhibitory mechanisms.

## Figures and Tables

**Table 1:** Comparison of inhibition constants between D-(−)-arabinose and other inhibitors of mixed-type inhibition for tyrosinase.

Inhibitor	*K*_*i*_ (mM)	Inhibitor	*K*_*i*_ (mM)
N-Benzylbenzamides [13]	1.3 × 10^−3^	5-Methoxysalicylic acid [14]	5.45
L-(−)-arabinose [15]	0.22	Terephthalic acid [16]	11.01
Isorhamnetin [17]	0.235	Isophthalic acid [18]	17.8
*o*-Toluic acid [19]	1.73	Phthalic acid [20]	65.84
Oxalic acid [21]	3.16	D-(−)-arabinose	210

## Abbreviations

DOPA: 3,4-Dihydroxyphenylalanine
ANS: 1-Anilinonaphthalene-8-sulfonate.
